# The Role of Mitochondria and Oxidative/Antioxidative Imbalance in Pathobiology of Chronic Obstructive Pulmonary Disease

**DOI:** 10.1155/2016/7808576

**Published:** 2016-12-26

**Authors:** Adam Jerzy Białas, Przemysław Sitarek, Joanna Miłkowska-Dymanowska, Wojciech Jerzy Piotrowski, Paweł Górski

**Affiliations:** ^1^Department of Pneumology and Allergy, 1st Chair of Internal Medicine, Medical University of Lodz, Łódź, Poland; ^2^Healthy Aging Research Centre (HARC), Medical University of Lodz, Łódź, Poland; ^3^Department of Biology and Pharmaceutical Botany, Medical University of Łódź, Łódź, Poland

## Abstract

Chronic obstructive pulmonary disease (COPD) is a common preventable and treatable disease, characterized by persistent airflow limitation that is usually progressive and associated with an enhanced chronic inflammatory response in the airways and the lung to noxious particles or gases. The major risk factor of COPD, which has been proven in many studies, is the exposure to cigarette smoke. However, it is 15–20% of all smokers who develop COPD. This is why we should recognize the pathobiology of COPD as involving a complex interaction between several factors, including genetic vulnerability. Oxidant-antioxidant imbalance is recognized as one of the significant factors in COPD pathogenesis. Numerous exogenous and endogenous sources of ROS are present in pathobiology of COPD. One of endogenous sources of ROS is mitochondria. Although leakage of electrons from electron transport chain and forming of ROS are the effect of physiological functioning of mitochondria, there are various intra- and extracellular factors which may increase this amount and significantly contribute to oxidative-antioxidative imbalance. With the coexistence with impaired antioxidant defence, all these issues lead to oxidative and carbonyl stress. Both of these states play a significant role in pathobiology of COPD and may account for development of major comorbidities of this disease.

## 1. Introduction

Chronic obstructive pulmonary disease (COPD) is characterized by persistent airflow limitation that is usually progressive and associated with an enhanced chronic inflammatory response in the airways and the lung to noxious particles or gases [1]. Although COPD is a common preventable and treatable disease, it is one of the major global health problems with 65 million people with moderate to severe stage of the disease and more than 3 million people died in 2005. The estimates show that COPD becomes the third leading cause of death worldwide in 2030 [2].

The major risk factor of COPD, which has been proven in many studies, is the exposure to cigarette smoke (CS) [3–8]. Numerous animal models of exposure to CS to induce the COPD-like anatomical changes were elaborated as well. Both mainstream [9–27] and second hand [28–51] exposure (mimicked by whole body exposure) models were described. Results of these experiments showed that exposing animals to CS allows inducing inflammatory response, small airway remodelling, excess mucus production, emphysema, and pulmonary hypertension, confirming the role of this factor in developing changes characteristic to COPD.

However, it is 15–20% of all smokers who develop COPD [52, 53]. There are also evidences that COPD may develop in nonsmokers [54–56]. Other factors, indicated as increasing the risk of development of COPD, are as follows: other than cigarette smoking form of tobacco use (e.g., cigar), marijuana, indoor pollution (such as from biomass cooking and heating in poorly ventilated dwellings), and occupational exposures to organic and inorganic dusts, chemical agents, and fumes [1]. Summarizing the contemporary knowledge, we should recognize the pathobiology of COPD as involving a complex interaction between several factors, including genetic vulnerability [57–62].

Numerous pathways are described to play a role in COPD pathogenesis. Oxidant-antioxidant imbalance is indicated as one of involved mechanisms [63]. The increased burden of oxidants in CS and increased amounts of reactive oxygen species (ROS) released from leukocytes and macrophages participating in inflammatory response in COPD are the sources of increased oxidative stress [64]. Oxidative stress is persisting after the cessation of CS, probably due to the continued production of reactive oxygen species from endogenous sources [65]. Increased level or prolonged exposure to ROS may lead to the pathological modification of nucleic acids, proteins, carbohydrates, or lipids and, as an effect, leads to changes in cellular metabolism.

Comorbidities of COPD are other serious diseases and chronic medical conditions that affect patients with COPD [66]. More than 30% of patients have one additional chronic disease, and another 40% have two or more comorbidities [67, 68]. The frequency of comorbidities increases with age [69]. Comorbidities severely impact costs of health care, intensity of symptoms, and quality of life and, most importantly, may contribute to life span shortening. Comorbid diseases are the risk factors of short- and long-term unfavourable prognoses [70]. Based on multiple studies, hypertension, ischemic heart disease, heart failure, pulmonary hypertension, diabetes mellitus, metabolic syndrome, gastroesophageal reflux disease, osteoporosis, anxiety, and depression may be indicated as the most prevalent comorbidities [71–90].

Mitochondria are multifunctional cellular organelles, which play an important role in numerous aspects of cell morphology and physiology, such as synthesis of adenosine triphosphate- (ATP-) intracellular transfer of energy, redox homeostasis, regulation of cellular metabolism [91], cell's calcium homeostasis, synthesis of steroids [92], and apoptosis [93, 94]. Mitochondria play also a role in innate immune system [95]. Generation of reactive oxygen and nitrogen species (RNS) is the part of normal functioning of mitochondria [63]. These organelles are the main source of production of ROS in eukaryotic cell and, consequently, developed compound antioxidant defence. Disturbances in antioxidant defence play an important role in oxidant-antioxidant imbalance. Numerous factors associated with suppression of antioxidant defence in lungs of patients with COPD were described [65].

The mechanisms by which mitochondria could contribute to COPD are still under investigation; however the present state of knowledge let us draw and analyse some pathways in pathophysiology of this disease.

## 2. Oxidative Stress in the Pathobiology of COPD

Due to respiratory physiology and anatomical conditions, our lungs are directly exposed to about 8,000 litters of air daily, which contain oxygen, numerous pathogens, pollutants, or allergens. All of the abovementioned have a potential to induce oxidative stress [96–99]. This is why development of efficient antioxidant defensive strategies was evolutionally necessary. In healthy cells antioxidant defences are able to efficiently limit the impact of ROS in disturbing homeostasis. However, adding other sources of reacting oxygen and nitric species, especially in presence of disturbances in antioxidant defence, may significantly contribute to oxidant-antioxidant imbalance and generate numerous harmful consequences.

For the purposes of our considerations, we used cigarette smoke exposure as the start point of analysed pathophysiological pathways. It is the risk factor, whose role has been well proven in the development of COPD and allowed to place the considerations in a strong theoretical background.

The gas phase of tobacco smoke contains about 10^15^ free radicals per puff [100]. Superoxide (O_2_^•−^) and hydroxyl (^•^OH) radicals are in high concentrations [101]. The first barrier in lungs, which is achieved by CS, is epithelial lining fluid (ELF), which covers airway epithelial cells (AECs). CS reacts with antioxidants in ELF. The most important antioxidant in ELF of the normal human lower respiratory tract is catalase. The others are the following: superoxide dismutase (SOD), glutathione reductase, and peroxidase, and ceruloplasmin [102]. After overcoming the ELF barrier, ROS in gaseous-phase of CS reach AECs plasma cell membranes, causing further pathological effects.

**Figure 1:**
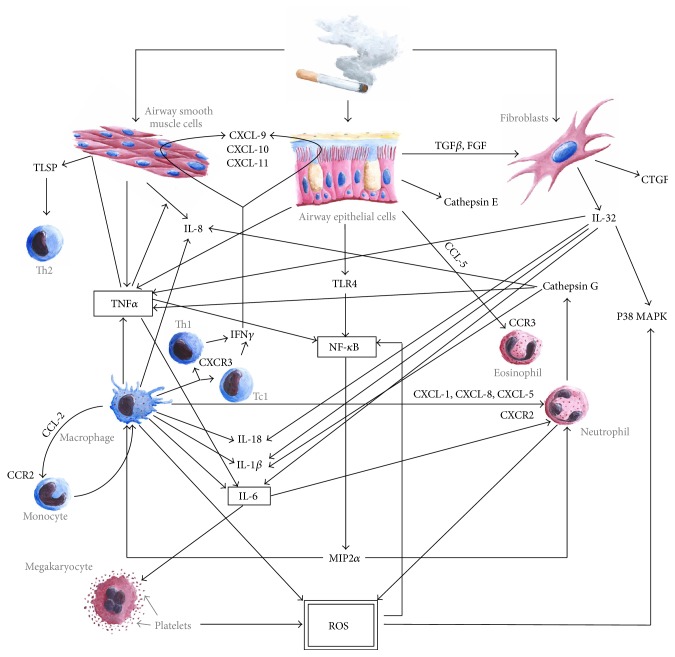
Selected pathways in the net of immunological interactions and ROS production in COPD.

The oxidative/antioxidative balance disturbances in COPD cannot be analysed separately from the sophisticated net of dynamic interactions in immunopathology of COPD (Figure 1). To depict it more clearly, we will analyse influence of airway tissues and immune cells separately.

### 2.1. Airway Tissues

The ROS in CS react with AECs plasma cell membranes, causing direct injury [101]. A class of lipids critically important for integrity of cellular membranes are glycerophospholipids. Oxidation of esterified unsaturated fatty acids changes biological activities of this lipids [103]. High levels of oxidized phospholipids may directly or indirectly engage Toll-like receptor (TLR) signalling, leading to lung injury. Stimulation of TLR4 can trigger the activation of pathway, which leads to activation of NF-*κ*B [104]. NF-*κ*B is a family of seven transcription factors, p50, p52, p100, p105, RelA/p65, RelB, and C-Rel. It plays a central role in inflammation and cell response by controlling gene network expression [105, 106] and has a central role in airway inflammation in COPD [107]. The members of this family can activate expression of many proinflammatory genes which play a role in lung inflammation as well [105, 108]. Activation of NF-*κ*B is redox-sensitive and can be regulated by the changes in the oxidant/antioxidant balance [105]. Di Stefano et al. reported an increased expression and activation and its correlation with airflow limitation in patients with COPD. Authors indicate a prominent role for epithelial cells in all smokers, as a source of NF-*κ*B protein [109]. NF-*κ*B is a target for acetylation and deacetylation [110, 111]. Although NF-*κ*B is not a histone protein, it can be targeted by the enzymes that regulate histone acetylation/deacetylation balance—histone acetyltransferases (HATs) and histone deacetylases (HDACs). In this way, these enzymes may regulate NF-*κ*B dependent proinflammatory gene transcription [105, 112]. While HATs activity in lungs of patients with COPD seems to be not altered, decreased total HDAC activity in samples of peripheral lung tissue, alveolar macrophages, and bronchial-biopsy specimens from patients with COPD has been reported [113]. This situation has a harmful clinical consequences, because of significant functions of HDACs in lung physiology and pathology: HDAC3, 5, and 8 are involved in the cell cycle, cell differentiation, and apoptosis, whereas HDAC2 plays a role in suppression of NF-*κ*B-mediated inflammatory gene expression by corticosteroids [114]. Deprivation of HDAC2 activity may also lead to loss of nuclear erythroid-2-related factor 2 (Nrf2) activity, which regulates gene expression of many antioxidant and cytoprotective genes [115]. Oxidative stress plays an important role in both activation of HATs (via activation of I*κ*B kinase *α*, NF-*κ*B inducing kinase and mitogen- and stress-activated protein kinase-1) and reducing the activity of HDAC2 via posttranslational modification and kinase-dependent signalling mechanisms [111]. Another important factor playing an important role in pathogenesis of COPD, secreted by epithelial cells after CS stimulation, is cathepsin E.

Airway smooth muscle cells (ASMCs) also play an important role in this net of cytokines and oxidative/antioxidative balance. CS can directly activate ASMCs to release IL-8 and enhances the release of IL-8 induced by TNF*α*. The effect of CS on IL-8 synthesis is associated with enhanced IL-8 gene transcription and increased expression of heme oxygenase-1 (HO-1). HO-1 is highly indictable by oxidative stress, which confirms its role in this process [116]. Other cytokines released by ASMCs are as follows: IL-6 [117, 118], CXCL-1 [119], CCL-2 (MCP-1) [120], neutrophil activating protein (NAP-2), epithelial neutrophil activating peptide 78 (ENA-78, also known as CXCL-5) [121], and CCL-17 (TARC) [122]. ASMCs stimulated by TNF*α* and IL- 1*β* can release thymic stromal lymphopoietin (TSLP), which triggers dendritic cell-mediated Th2 response [123].

Both AECs and ASMCs release CXCL-9, CXCL-10, and CXCL-11 following stimulation with IFN*γ* [124–127]. This effect can be augmented by the synergistic interactions of IFN*γ* with TNF*α* [124, 128], which can also be secreted by both of these cells [129].

Fibroblasts are the main cell type in the lung interstitium [130], are essential for development [131], and form the architecture of alveoli [132]. CS induces ROS-dependent nucleic acid oxidation in alveolar fibroblasts, which may play a role in the pathogenesis of emphysema. Authors reported significant oxidation of nucleic acids localized to alveolar lung fibroblasts, increased levels of 8-oxoguanine-DNA glycosylase mRNA expression, and decreased concentrations of endonuclease III homologue 1, single-strand-selective monofunctional uracil-DNA glycosylase 1, and Y-box binding protein 1 mRNA in lung samples obtained from subjects with very severe COPD compared with COPD in stages 2-3 (study was published in 2010 and this is why authors used old GOLD classification of severity of the disease) or no-COPD [132]. There are evidences that CS induces NF-*κ*B mobilization to the nucleus in human lung fibroblasts, may induce cyclooxygenase-2 and microsomal prostaglandin E synthase synthesis, and increases production of prostaglandin E [133].

### 2.2. Immune Cells

Rtp801 may act as a potential amplifying switch in the development of cigarette smoke-induced lung injury, leading to emphysema. The upregulation of Rtp801 expression by CS in the lung relied on oxidative stress-dependent activation of the CCAAT response element. Rtp801 activates NF-*κ*B, amplifying inflammatory and cell death responses [134]. NF-*κ*B activation may lead to expression of MIP-2 *α*, which is a primary CXC chemokine that can cause neutrophil chemotaxis and activation [135]. Rtp801 enhanced lung macrophage infiltration as well [134]. In acute exposure to CS, Rtp801 plays a role in alveolar cell apoptosis, which further promotes inflammation and oxidative stress [136].

As we mentioned above, Rtp801 plays a role in creating neutrophil and macrophage infiltration in lung tissue. Macrophages secrete tumour necrosis factor alpha (TNF*α*), interleukin 8 (Il-8), which is the major neutrophil chemotactic factor in lungs [137], leukotriene B4 (LTB4), monocyte chemotactic protein 1 (MCP-1), matrix metalloproteinases 2, 9, and 12 (MMP-2, MMP-9, and MMP-12), and cathepsins K, L, and S and generate ROS [129, 138].

Metalloproteinases (MMPs) are proteolytic enzymes, which play roles in many physiological processes, including morphogenesis, cell migration, and angiogenesis [139]. Neutrophils secrete MMP-8, MMP-9, and serine proteases (neutrophil elastase, proteinase, and cathepsin G) [138].

The research of the impact of CS-induced oxidative stress on NF-*κ*B activation in human lymphocytes revealed significance of reactive nitrogen species in this process. NF-*κ*B activation was dependent on intracellular formation of peroxynitrite from CS-derived nitric oxide [140]. Moreover, products of oxidation may contribute to deepening oxidative stress; for example, oxidized palmitoyl-arachidonoyl-phosphatidylcholine may increase intracellular and extracellular levels of superoxide radical [103, 141, 142].

TNF*α* is one of the crucial cytokines which plays a role in the development of airway inflammation [143]. TNF*α* and IL-1 could stimulate ROS production and are the agonists activating NF-*κ*B [105, 129, 144]. Inducing NF-*κ*B and AP-1 in human lung, TNF*α* could play a role in the expression of IL-6 [145, 146]. IL-6 acts to increase the synthesis of acute phase proteins, including CRP, but also affects the levels of platelets and neutrophils by stimulation of the differentiation of megakaryocytes to platelets, possible stimulation of thrombopoietin production, and involvement in recruitment of neutrophils [147–151]. In various circumstances, actively participating in inflammation, platelets play a role as an immune cells [152]. However, for our considerations more important is their role in oxidative/antioxidative balance. It has been proven that platelets themselves have the ability to produce ROS [153]. This issue may have systemic consequences; via an oxidative stress-mediated mechanism involving gp91phox activation, platelets modify low density lipoprotein (LDL), possibly contributing to the progression of atherosclerotic disease [154].

Another issue is that TNF*α* may induce IL-32 mRNA expression and protein release from primary human lung fibroblasts via the activation of Jun N-terminal kinase and Akt signalling pathways [143]. An increased expression of IL-32 in lung tissue of patients with COPD and its correlation with the degree of airflow obstruction was reported. Moreover, IL-32 seems to be involved in the corticosteroid resistance of COPD inflammation [155]. IL-32 induces the production of IL-1*β*, TNF*α*, IL-6, and IL-18 in alveolar macrophages [155, 156]. IL-32 induct abovementioned proinflammatory cytokines via activation of NF-*κ*B and p38 mitogen-activated protein kinase (p38 MAPK) signalling pathways [143, 157].

LRB4 seems to be the major factor in producing ROS and RNS in myeloid cells, including neutrophils and macrophages [158, 159]. LRB4 involves ROS and RNS production via activation of NF-*κ*B and MAPKs [160].

MCP-1 is a member of the C-C chemokine family and a potent chemotactic factor for monocytes [161]. It can be secreted not only by macrophages, but also by smooth muscle cells, endothelial cells, and fibroblasts. There are evidences that MCP-1 is a chemoattractant for arterial smooth muscle cells (SMC) as well, and ROS seems to play a role in MCP-1 stimulated SMC migration in a positive activation loop, which, in turn, may account for the role played by MCP-1 in the development and progression of atherosclerosis [162].

Protease-antiprotease imbalance in lungs plays an important role in the pathogenesis of CS-induced emphysema. One of proteases, which are significantly involved in COPD pathogenesis, is cathepsins. The abovementioned cathepsins, secreted by neutrophils and macrophages, are cysteine (K, L, and S) or serine (G) proteases [163, 164]. Besides proteolytic activity, cathepsin G interacting with other cytokines contributes indirectly to ROS production. Cathepsin G, secreted by neutrophils, enhances the activity of IL-8 [165] and is able to activate IL-1beta and TNF-alpha [166]. Moreover, by proteolytic cleavage of phospholipid transfer protein, cathepsin G may enhance the injurious inflammatory responses in COPD [167].

Another curious issue is that ROS may play a role in the cough reflux. Cough is one of the most recognizable symptoms of COPD. Oxidative stress may evoke profound effects on airway afferent nerves, particularly through the modulation of neuronal transient receptor potential channels [168].

## 3. Mitochondrial-Derived ROS and COPD

The continued presence of oxidative stress in COPD most likely arises from endogenous sources [65]. One of endogenous sources of ROS is mitochondrial respiration. Mitochondrial electron transport chain, even under normal conditions, may “leak” 1-2% of all electrons as ROS [169, 170]. There are some intra- and extracellular factors, which may increase this amount and significantly contribute to oxidative-antioxidative imbalance.

As we have described above, CS play an important role in oxidative-antioxidative imbalance. However, there are strong evidences that gaseous-phase ROS are not able to diffuse through plasma membranes of AECs and, as an effect, enter the systemic circulation [101]. The answer to the question about the systemic effects of CS seems to be rather in the lipophilic components, including polycyclic aromatic hydrocarbons, aldehydes, amines, heavy metals, or phenolic compounds, which easily pass the cell membranes and enter the circulation. The crucial study for understanding the systemic effects of CS was published by van der Toorn et al. [101]. Authors reported that the lipophilic fraction present in CS extract is responsible for a decrease in mitochondrial membrane potential, ATP production, and concomitant generation of mitochondrial ROS. Authors provided evidence that functional electron transfer chain in mitochondria is essential in CS-induced ROS generation. The observations were compliant with previous proofs that blocking a functional electron transfer chain results in enhanced generation of ROS [171]. It is worth highlighting that these issues are not limited to airway cells, but also circulating cells may be affected in this way [172]. This knowledge gives a better insight to understand the systemic effects of CS and its contribution to oxidative/antioxidative balance disturbances.

There are some evidences that some cytokines play an important role in increasing production of mitochondrial-derived ROS. ASMCs from patients with COPD, when subjected to inflammatory stress from IL-1, IFN*γ*, and TNF*α*, produce larger amounts of mitochondrial-derived ROS [65].

Increasing evidence suggests a significant role of MMP-2 in damage of mitochondria. The loop of interactions seems to exist between MMP-2 and mitochondria: mitochondrial-derived ROS can drive MMP-2 activation, which may result in a negative feedback cycle that degrades mitochondrial membrane potential and impairs mitochondrial function [173]. Another study, conducted on adult rat ventricular myocytes, showed that inhibition of MMP-2 inhibits *β*-adrenergic receptor-stimulated activation of JNKs as well as cytochrome c release, suggesting the role of MMP-2 in activating JNK-dependent mitochondrial death pathway [174]. Moreover, destructive effects of MMPs seem to participate in the development of diabetic retinopathy: activated MMP-2 and MMP-9 can enter into the mitochondria, damage their structure and integrity, and as an effect release cytochrome c, activating apoptosis [175]. As an effect of mitochondrial damage, ROS levels continue to increase and begin to damage mtDNA. The damaged mtDNA continues to dysfunction the electron transport chain, which results in enhanced generation of ROS, closing the loop.

**Figure 2:**
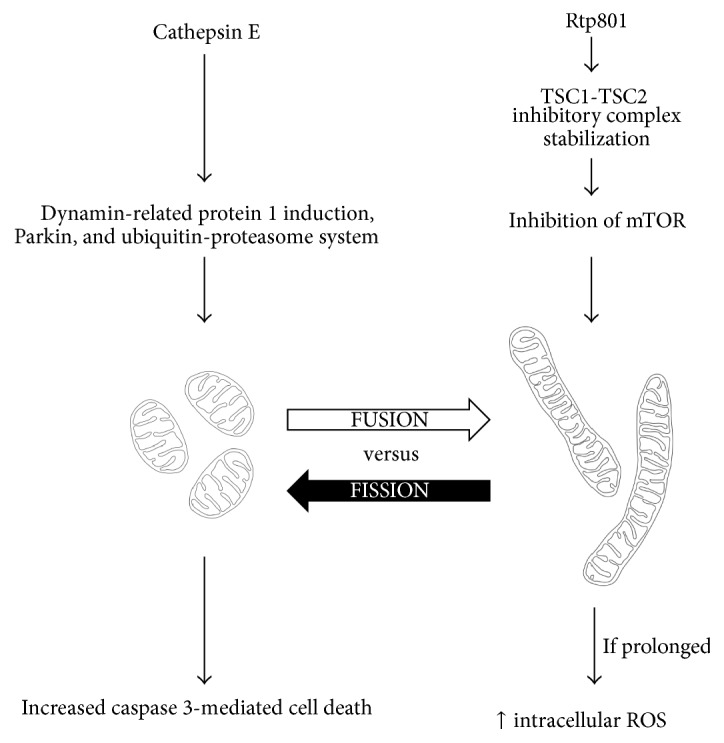
Hypothetical influence of cathepsin E and Rtp801 on mitochondrial fusion-fission balance.

We have mentioned role of cathepsins in oxidative-antioxidative imbalance. One of them, cathepsin E (CE), may directly influence mitochondrial function. Human lung sections from patients with COPD indicated increased expression of CE protein in the lung epithelial cells [176]. Unlike other members of cathepsin family, CE has not been reported to exhibit proteolytic activity. CE overexpression seems to lead to pulmonary emphysema by increasing mitochondrial fission via dynamin-related protein 1 induction, Parkin, and ubiquitin-proteasome system, thereby influencing the mitochondrial fusion-fission balance, leading to increased caspase 3-mediated cell death (Figure 2) [176].

Above, we have described the role of CS in Rtp801 expression. Rtp801 may also link oxidative stress, with the activation of immune system via inhibition of mammalian target of rapamycin (mTOR) by stabilizing the TSC1-TSC2 inhibitory complex [134]. mTOR is an important signalling hub which plays a role in cell growth regulation, nutrient metabolism, protein synthesis, mRNA translation, cell motility, survival, and autophagy [177–181]. It is known that pharmacological and genetic inhibition of mTOR leads to increased mitochondrial fusion [182]. It could be a protective mechanism as mitochondrial fusion can protect cells from harmful effects of mitochondrial DNA (mtDNA) mutations by allowing functional complementation of mtDNA gene products [183]. Moreover, during autophagy, elongated mitochondria are spared from autophagic degradation, possess more cristae, increased levels of dimerization and activity of ATP synthase, and maintain ATP production [184]. On the other hand, prolonged elongated mitochondria may result in higher production of intracellular ROS and lower activity of mitochondrial respiration, which as an effect ultimately leads to cellular senescence (Figure 2) [185]. Recently, premature senescence is considered as an important factor in pathobiology of COPD [186].

The ETC, mitochondrial membrane permeability, and apoptotic signalling all seem to be affected in COPD: increased cytochrome oxidase activity and citrate synthase in the vastus lateralis muscle and upregulation of activities of other membrane-bound respiratory chain enzymes and abnormal mitochondrion permeability transition pore kinetics and cytochrome c release in skeletal and respiratory muscles in patients with COPD were reported [187–190].

## 4. Suppression of Antioxidant Defense in COPD

As we can see, majority of already known elements of the net of dynamic interactions in immunopathology of COPD play a role in oxidant-antioxidant balance. The antioxidant defence of healthy cells is able to efficiently limit their impact in disturbing homeostasis, whereas suppression of antioxidant defence may significantly shift the equilibrium in oxidant-antioxidant balance to the side of oxidants.

The principal antioxidant in the lung is glutathione (GSH) [130]. The rate-limiting enzyme in de novo GSH synthesis is gamma-glutamylcysteine synthetase. Cell-specific expression or regulation of gamma-glutamylcysteine synthetase may play an important role both in the defence against oxidants and in the pathogenesis of oxidant-associated airway diseases. The expression of heavy subunits of gamma-GCS in the central bronchial epithelium showed a tendency to be higher in nonsmokers compared with smokers and alveolar macrophages of nonsmokers had higher levels of heavy and light subunits of gamma-GCS-HS than did smokers. The expression of heavy subunits gamma-GCS in the central bronchial epithelium was more marked in nonsmokers than in patients with COPD and smokers. The tendency to decrease subunits of gamma-GCS in cigarette smokers may further predispose lung cells to ongoing oxidant stress. Finally, it contributes to the progression of lung injury [191].

Decrease of mRNA expression for catalase, glutathione S-transferase P1, glutathione S-transferase M1, microsomal epoxide hydrolase, and tissue inhibitor of metalloproteinase 2 in COPD lung tissues was also reported [192].

Principal antioxidant within the mitochondria and responsible for degrading superoxide (O_2_^•−^) into H_2_O_2_ is manganese-SOD (MnSOD) [193]. Expression of this superoxide dismutase and catalase in ASMCs can be inhibited by transforming growth factor (TGF-*β*) [194]. Both catalase and MnSOD are under the control of Forkhead box class O 3a (FoxO3), which significantly decreased in lungs of smokers and patients with chronic obstructive pulmonary disease, as well as in lungs of mice exposed to CS. Moreover, genetic ablation of FoxO3 led to pulmonary emphysema and exaggerated inflammatory response in lungs of mice exposed to CS. Hwang et al. reported that CS induced the translocation of FoxO3 into the nucleus where FoxO3 interacted with NF-*κ*B and disrupted NF-*κ*B DNA-binding ability, leading to inhibition of its activity [195]. Authors presented also that targeted disruption of FoxO3 resulted in downregulation of antioxidant genes in mouse lungs in response to CS exposure. These evidences support the hypothesis about the role of deficiency of FoxO3 in development of COPD/emphysema.

Another important transcription factor in antioxidant regulation is nuclear erythroid-2-related factor 2 (Nrf2). Nrf2 plays a significant role in cellular defence against oxidative stress by inducing the expression of many antioxidant genes. Mercado et al. reported that where high levels of oxidative stress occur, reduction of Nrf2 activity can be observed. Authors hypothesized that Nrf2 instability may be caused by downregulation of histone deacetylase 2 (HDAC2). They concluded that reduced HDAC2 activity in COPD may account for increased Nrf2 acetylation, reduced Nrf2 stability, and impaired antioxidant defences [196].

Increasing evidence suggests importance of differential expression of the aryl hydrocarbon receptor (AhR) in downregulation of expression of antioxidant enzymes expression. AhR plays a role in attenuating pulmonary inflammation caused by cigarette smoke [197]. AhR expression influences the levels of basal MnSOD and CuZn-SOD. AhR may also regulate both apoptosis and proliferation in the lung, which has important implications for the development of COPD [193].

## 5. Carbonyl Stress and COPD

Carbonyl stress can be defined as accumulation of reactive carbonyl species and subsequent protein carbonylation. Reactive carbonyl species are formed as the effect of oxidation of carbohydrates, lipids, DNA, RNA, and proteins [65, 198]. Carbonyl stress causes nonenzymatic posttranslational modifications on proteins that may alter their function, as well as lead to forming of danger-associated molecular patterns and neoautoantigens.

**Figure 3:**
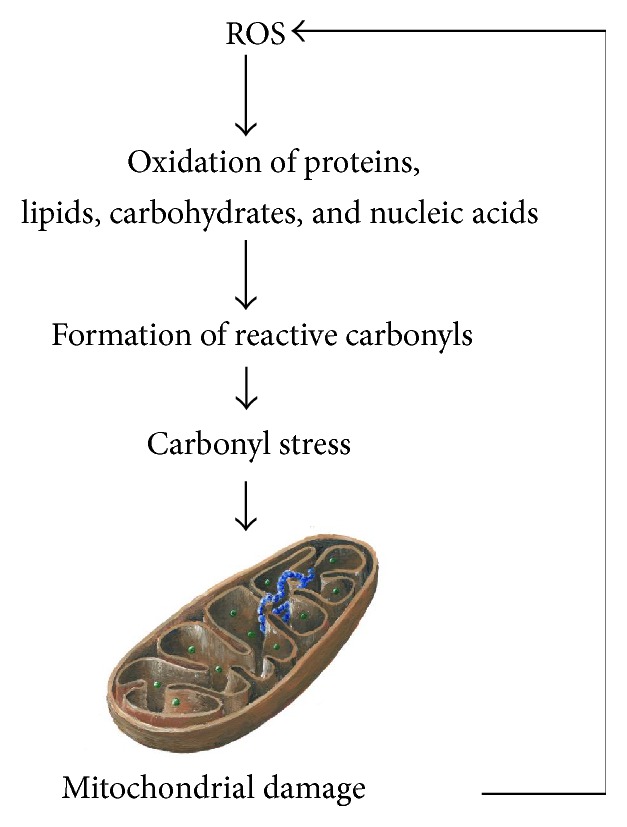
Damage of mitochondrial proteins by carbonyl stress leads to enhanced endogenous ROS production by the damaged mitochondria, forming the amplification loop in relationships in “oxidative stress, mitochondria, and carbonyl stress axis.”

Proteins can be carbonylated directly (“primary protein carbonylation”), by direct oxidation of side chains of amino acids residues, or indirectly (“secondary protein carbonylation”), in reaction via the addition of aldehydes such as those generated from lipid peroxidation processes [199]. Numerous examples of protein carbonylation effects were reported. Carbonylated creatine kinase may contribute to the loss of muscle function associated with age and disease [198]. Barreiro et al. assessed the levels of protein oxidation, lipid peroxidation, catalase and Mn-SOD expressions, nitric oxide synthases, and protein tyrosine nitration in quadriceps muscles of patients with patients with COPD, finding development of both oxidative and nitrosative stresses in the quadriceps of patients with COPD, suggesting their involvement in muscle dysfunction [200]. Moreover, damage of mitochondrial proteins by carbonyl stress leads to enhanced endogenous ROS production by the damaged mitochondria [65] forming the amplification loop in relationships in “oxidative stress, mitochondria, and carbonyl stress axis” (Figure 3).

Malondialdehyde (MDA) is a major product of lipid peroxidation. Its increased levels in the lungs of patients with COPD and their association with disease severity were reported [201]. Another product of lipid peroxidation, 4-hydroxy-2-nonenal (4-HNE) is a key mediator of oxidant-induced cell signalling and apoptosis. Rahman et al. observed a positive correlation between 4-HNE adducts and TGF-*β*1 protein and mRNA, as well as gamma-GCS mRNA levels in airway and alveolar epithelium, and a significant inverse correlation between the levels of 4-HNE adducts in alveolar epithelium, airway endothelium, and neutrophils and forced expiratory volume in one second (FEV_1_).

4-HNE also exerts an important inhibitory influence on mitochondrial functions [202].

Carbonyl stress is also blamed for pathophysiologic mechanisms associated with the development of COPD, such as bronchial constriction, tissue remodelling, impaired steroid function, mucus hypersecretion, impaired phagocytic function, proinflammatory signalling, and induction of autoimmunity [65].

Recent state of knowledge led us to link carbonyl stress with numerous diseases.

One of major groups of diseases associated with carbonyl stress is cardiovascular disorders. Yavuzer et al. suggested that oxidative stress may influence both the development and progression of aging and hypertension [203]. Tanito et al. reported enhanced expressions of 8-hydroxy-2′-deoxyguanosine and protein carbonylation in aorta, heart, and kidney from spontaneously hypertensive rats and stroke-prone spontaneously hypertensive rats compared with Wistar-Kyoto rats, concluding that redox imbalance in essential organs may play a crucial role in the development and pathogenesis of hypertension [204].

Hoshino et al. demonstrated impaired mitochondrial AAA+ protease activity in pressure overload heart failure in mice. The expression of AAA+ proteases was not decreased and genetic antioxidant intervention in mitochondria recovered proteolytic activity, which suggested that the oxidative posttranslational modifications of AAA+ proteases may have been responsible for decreasing proteolytic activity. The decline in AAA+ protease activity was closely associated with decreased protein turnover and increased oxidative damage in the electron transport chain, leading to mitochondrial respiration deficiency and left ventricular contractile dysfunction [205].

Steinberg reported that 4-HNE- and MDA-modified LDLs are directly involved in the mechanisms of fatty streak formation, an early step of atherogenesis [206]. In addition to that, reactive carbonyl compounds seem to also contribute to aggregation and dysfunction of fibrinogen. Its deposition on blood vessel walls plays an important role in the pathology of atherosclerosis as well [207].

Emelyanova et al. observed higher levels of 4-HNE in patients with atrial fibrillation [208].

Hopps et al. examined lipid peroxidation and protein oxidation in patients with obstructive sleep apnoea, finding a significant correlation with the severity of the disease. Authors observed a significant correlation between protein oxidation and apnoea/hypopnea index values, a positive correlation between carbonyl groups and ODI, and a negative correlation between carbonyl groups and mean oxygen saturation [209].

Carbonyl stress is also a contributing factor to pathogenesis of metabolic syndrome, chronic complications associated with diabetes and renal failure [210–212]. There is an evidence that 4-HNE could contribute to the mechanisms of obesity and insulin resistance, via the modification of adipose regulatory proteins [213]. Furukawa et al. reported that increased oxidative stress in accumulated fat is an early instigator of metabolic syndrome and that the redox state in adipose tissue may be useful therapeutic target for obesity-associated metabolic syndrome [214].

There is also an evidence that the deposition of 4-HNE and MDA adducts in the lens of aged rats or submitted to high oxidative stress is correlated with the apoptosis of lens cells and cataract formation [215].

As we can see, oxidative/carbonyl related diseases figured out above, cover also the most prevalent comorbidities of COPD. Therefore, we can hypothesize that oxidative/carbonyl stress may be considered as a link between COPD and its comorbidities.

## 6. Conclusions

Numerous exogenous and endogenous sources of ROS present in pathobiology of COPD are associated with its presence in CS, effects of lipophilic components of CS, and sophisticated net of dynamic interactions in inflammatory process characteristic for this disease. One of endogenous sources of ROS is mitochondria. Although leakage of electrons from ETC and forming of ROS are the effect of physiological functioning of mitochondria, there are various intra- and extracellular factors, which may increase this amount and significantly contribute to oxidative-antioxidative imbalance, what with impaired antioxidant defence, deepen this state, and contribute to development of oxidative stress. Oxidation of carbohydrates, lipids, DNA, RNA, and proteins may lead to reactive carbonyl species formation. Their accumulation, known as carbonyl stress, seems to be responsible for harmful consequences for cellular metabolism. As an effect, carbonyl stress contributes significantly to pathobiology of COPD and may account for development of major comorbidities of this disease.
